# The effect of cocaine on gastric mucosal PGE_2_, LTC_4_ and ulcerations

**DOI:** 10.1155/S096293519300050X

**Published:** 1993

**Authors:** L. D. G. Angus, T. A. Stein, L. J. Auguste, J. Gintautas, J. Westervelt, L. Wise, G. W. Shaftan

**Affiliations:** 1 Department of Surgery The Brookdale Hospital Medical Center Brooklyn NY 11212 USA; 2 Department of Surgery The Long Island Jewish Medical Center New Hyde Park NY 11042 USA

## Abstract

The association between cocaine use and acute gastroduodenal perforation is known. The effect of cocaine and stress on gastric mucosal ulceration and the levels of prostaglandin E_2_ (PGE_2_) and leukotriene C_4_ (LTC_4_) was studied in 40 Sprague–Dawley rats. Controls received intraperitoneal (i.p.) saline, ten received i.p. cocaine (35 mg/kg), ten were stressed by the cold restraint method, and ten had i.p. cocaine and stress. Cocaine alone did not induce ulceration, but decreased PGE_2_ levels. Stress alone caused ulceration, but was not associated with a change in either PGE_2_ or LTC_4_ levels. When combined with stress, however, cocaine caused a three-fold increase in ulceration and a significant increase in PGE_2_ and LTC_4_ levels. Stress may predispose the cocaine addict to loss of gastroduodenal mucosal integrity, which is related to an imbalance of PGE_2_ and LTC_4_ synthesis.


Mediators of Inflammation 2, 363–365 (1993)

### The effect of cocaine on gastric mucosal $PGE_2$, $LTC_4$ and ulcerations

L. D. G. Angus,[1,CA] T. A. Stein,[2] L. J. Auguste,[2] J. Gintautas,[1] J. Westervelt,[1] L. Wise[2] and G. W. Shaftan[1]

[1]Department of Surgery, The Brookdale Hospital Medical Center, Brooklyn, NY 11212; [2]Department of Surgery, The Long Island Jewish Medical Center, New Hyde Park, NY 11042, USA

[CA] Corresponding Author

**THE association between cocaine use and acute gastroduodenal perforation is known. The effect of cocaine and stress on gastric mucosal ulceration and the levels of prostaglandin $E_2$ ($PGE_2$) and leukotriene $C_4$ ($LTC_4$) was studied in 40 Sprague–Dawley rats. Controls received intraperitoneal (i.p.) saline, ten received i.p. cocaine (35 mg/kg), ten were stressed by the cold restraint method, and ten had i.p. cocaine and stress. Cocaine alone did not induce ulceration, but decreased $PGE_2$ levels. Stress alone caused ulceration, but was not associated with a change in either $PGE_2$ or $LTC_4$ levels. When combined with stress, however, cocaine caused a three-fold increase in ulceration and a significant increase in $PGE_2$ and $LTC_4$ levels. Stress may predispose the cocaine addict to loss of gastroduodenal mucosal integrity, which is related to an imbalance of $PGE_2$ and $LTC_4$ synthesis.**

**Key words:** Cocaine, Leukotriene $C_4$ ($LTC_4$), Prostaglandin $E_2$ ($PGE_2$), Ulcerations

### Introduction

The illicit use of cocaine continues to be a significant social problem. It is estimated that 30 million Americans have used cocaine, five million use it regularly and each day another five thousand use it for the first time.[1–3] A particularly serious form of such abuse is the use of a free base form of cocaine commonly referred to in our inner city streets as 'crack'. The substance is generally prepared from cocaine by heating with sodium bicarbonate and water in glass vials and that, in turn, makes the substance resistant to destruction and, therefore, administered effectively by smoking. Blood levels can be achieved which are similar to those after intravenous dosing.[4]

Cocaine is a very strong stimulant of the central nervous system. Because of its high addiction potential, 'crack' has not only worsened the drug abuse problem but has also created a number of potentially lethal complications including stroke, intestinal ischaemia, diffuse alveolar haemorrhage, pneumomediastinum, pneumopericardium, pneumonia, myocardial infarction as well as muscle and skin necrosis.[5–11]

A number of investigators have suggested that gastroduodenal perforations may indeed be another serious complication of 'crack' abuse.[12–15] The cause of gastroduodenal ulcer perforation in 'crack' cocaine users is not known; however, the toxic effect may be related to its potent vasoconstrictive action leading to reduced blood flow to the stomach and duodenum.[16] Stress induced gastric mucosal ulcerations appear to be related to a decrease in mucosal prostaglandin $E_2$ ($PGE_2$), a potent vasodilator, and an increase in synthesis of leukotriene $C_4$ ($LTC_4$), a potent vasoconstrictor.[17] Numerous factors have been shown to affect the gastric mucosal $PGE_2$ and $LTC_4$ levels and, hence, the incidence of ulcer formation.[18,19] This study was undertaken to determine whether the effect of cocaine on the gastric mucosa was mediated by changes in $PGE_2$ and $LTC_4$ synthesis.

### Materials and Methods

Forty male Sprague–Dawley rats weighing 125–150 g were kept in our animal facility at room temperature in cages with wide wire bottoms to prevent coprophagia. The animals were maintained according to the principles given by the Institute of Laboratory Animal Resources and all protocols were approved by our institutional review boards.[20] The animals were fed standard rat chow and after 1 week of conditioning, they were allocated into four equal groups. All animals were deprived of food 24 h prior to the experiment; however, all were given water *ad libitum*. Group I rats ($n = 10$) had 1 ml of 0.9% saline injected intraperitoneally (i.p.) and were not subjected to stress. Group II animals ($n = 10$) were subjected to stress and received no intraperitoneal injections. Groups III animals ($n = 10$) received intraperitoneal cocaine at 35 mg/kg but were not subjected to stress. Group IV rats ($n = 10$), however, received cocaine i.p. and

© 1993 Rapid Communications of Oxford Ltd were also subjected to stress according to a modification of the cold restraint model as described by Brodie and Hanson.[21] The cold restraint stress method consisted of immobilization under a tight wire screen cage for 2 h at room temperature and then at 4°C for an additional 2 h. At the end of the stress period, all animals were sacrificed by cervical dislocation. The unstressed animals were kept for 4 h at room temperature prior to sacrifice. A cocaine dose of 35 mg/kg was selected because the $LD_{50}$ of i.p. cocaine has been reported at 35 mg/kg.[22] Immediately after sacrifice, the stomachs were excised and opened along the lesser curvature. The mucosa was rinsed with iced normal saline solution and any ulcerations present were counted using loops of 2.5 × magnification. The gastric mucosa was then stripped, minced and weighed. Between 125 and 175 mg of tissue as suspended in 1 ml of 0.1 M NaCl, 4.8 mM KCl, 1.2 mM $K_2PO_4$, 5 mM glutathione, 1 mM hemine, 3 mM $MgSO_4$, 5 mM poloxamer 188 and 8.8 mM HEPES buffer at pH 8.0. After 1 min, the tissue suspension was centrifuged at 15 000 × *g*. The supernatant was decanted and the pellet was resuspended in 1 ml of the poloxamer buffer for 1 min to allow for the synthesis of $PGE_2$ and $LTC_4$ from endogenous arachidonic acid. The reaction was terminated by the addition of 1.5 ml of acetone at −20°C. Prostaglandin $E_2$ and leukotriene $C_4$ were extracted and quantitated by reversed-phase high pressure liquid chromatography (HPLC).[23,24] The rate of synthesis was calculated in units of μg/g/min. The means and S.E.M. were calculated and the differences between the groups were determined by ANOVA with statistical significance being inferred for a $p$ value $<0.05$.

*Plasma cocaine concentrations:* Serum levels of cocaine were also obtained in order to correlate the peak plasma concentration with the physiological changes noted in the gastric mucosa since the duration of stress was 4 h.

Thirty male Sprague–Dawley rats were administered 35 mg of cocaine HCl in water intraperitoneally. Six animals were sacrificed at 10, 30, 60, 120 and 180 min. All samples contained 2.5 mg of sodium fluoride per ml of plasma to prevent hydrolysis of cocaine by cholinesterase. Cocaine was extracted and quantitated by reversed-phase HPLC.[25] A stock standard of cocaine as the free base was prepared in methanol at a concentration of 1 g/l and stored at −20°C until needed, to produce a standard curve which ranged from 20 to 200 000 mg/ml.

### Results

Cold restraint stress induces significant lesions in the gastric glandular mucosa (Table 1). Mucosal erosions were haemorrhagic and varied from 1 cm to 1 mm in length. Some of the erosions in the group which had both stress and cocaine were true ulcers, which have been reported in another study to occur with stress.[26] After stress alone, the synthesis of $PGE_2$ and $LTC_4$ levels were not statistically different from control mucosal levels (Table 1). The administration of cocaine caused a 30% decrease in the synthesis of $PGE_2$, no change in $LTC_4$ synthesis, and no ulceration. However, with the addition of stress, cocaine caused significant mucosal injury (Group IV). The synthesis of $LTC_4$ in Group III was similar to that of the stress groups (Group II). When cocaine was given just before stress, the synthesis of both $PGE_2$ and $LTC_4$ were both increased. The number of lesions were also increased ($p < 0.05$) compared to that of the stressed group (IV *vs.* II).

**Table 1.** Mucosal injury and synthesis of $PGE_2$ and $LTC_4$

| Group | Treatment | Ulcers | $PGE_2$ (μg/g) | $LTC_4$ (μg/g) |
|---|---|---|---|---|
| I | Saline | 0 | 1.8 ± 0.1 | 3.5 ± 0.2 |
| II | Stress | 5.2 ± 1.9* | 1.6 ± 0.1 | 3.3 ± 0.1 |
| III | Cocaine | 0 | 1.3 ± 0.1* | 3.3 ± 0.1 |
| IV | Stress ± cocaine | 16.0 ± 3.1* | 2.7 ± 0.4* | 4.3 ± 0.5* |

* $p < 0.05$ compared to Group I.

*Cocaine levels:* Ten min after an i.p. injection of cocaine, plasma levels were 91 ± 20 ng/ml and at 30 min were 96 ± 24 ng/ml. At 60 and 120 min, the concentrations were 146 ± 37 and 143 ± 43 ng/ml, respectively. By 180 min, the levels fell to 79 ± 25 ng/ml.

### Discussion

Stress induction of gastric ulceration is probably related to the action of bioactive peptides, thyrotropin releasing hormone, vasoactive intestinal peptide, and gastrin, on the dorsal vagal complex in the brain to stimulate cholinergic pathways and increase gastric acid secretion.[27,28] Although $PGE_2$ synthesis was lower by 13% after stress, no statistically significant decrease occurred. In other studies, stress usually causes a 25% decrease in $PGE_2$ which is related to an inhibition of neurotensin on the central nervous system and peripheral adrenergic release.[26,29]

Cocaine stimulates the central nervous system and sympathetic pathways by releasing dopamine and inhibiting serotonin, epinephrine and norephinephrine uptake at neuronal synapses, resulting in vasoconstriction.[8] When cocaine was given alone (Group II) $PGE_2$ synthesis decreased by 34% but mucosal ulceration did not occur, suggesting that vasoconstriction was not sufficient to cause ischaemia.

When cocaine was injected in stressed rats (Group IV), significant ulceration occurred. The synthesis of $PGE_2$ and $LTC_4$ also increased which is related to the stimulation of both parasympathetic and sympathetic pathways. The mechanism for this increase in synthesis may be similar to that occurring in chronic colitis.[30] Stress-induced injury to the gastric mucosa appears to predispose cocaine stimulation of $LTC_4$ synthesis and that manifests itself as an exacerbation of ulceration on the gastric mucosa. The vasoconstriction caused by stress, namely the 'Flight or Fight' response, coupled by the powerful vasoconstrictive effect of $LTC_4$ appears to be enough to create a worsening of gastric ulcerations in the rat model. It is conceivable that the chronic use of cocaine in humans will produce gastric ulcerations and perforations in a mechanism mediated by the delicate balance between $PGE_2$ and $LTC_4$.

Cocaine was administered as the hydrochloride salt, because it is water soluble and appears to maintain nearly constant plasma concentrations for 3 h, when administered by intraperitoneal injection. The deleterious effects on the central nervous system require penetration of the blood–brain barrier by cocaine, which depends on plasma levels. Since the development of gastroduodenal lesions by stress requires approximately 4 h to develop, the time used to observe erosions induced by cocaine may have not been adequate in this study. The time, however, was sufficient to cause significant biochemical changes in the mucosa when cocaine was combined with stress. Although cocaine has been shown to protect against experimental gastric stress ulceration in rats, this study demonstrates that this effect is dose dependent.[31] The cytoprotective effect of cocaine seen at low dose seems to be lost when cocaine is administered at high doses.

In conclusion, when the integrity of the gastric mucosa is already compromised, the administration of a large dose of cocaine exacerbates mucosal injury leading to extensive ulceration via a mechanism of vasoconstriction and ischaemia probably mediated by an excessive production of $LTC_4$. Cocaine is a powerful sympathetic stimulant. Although the mechanism of ulceration appears to be intricately related to the delicate balance between $PGE_2$ and $LTC_4$, the effect of the drug on gastrointestinal blood flow in relation to ulcer disease needs to be investigated further as that may be the initial factor that triggers the imbalance between $PGE_2$ and $LTC_4$ leading to gastric mucosal ulcerations and eventually perforation.

### References

1. Abelson HI, Miller JD. A decade of trends in cocaine use in the household population. *Natl Inst Drug Abuse Res* 1985; **61**: 35–49.
2. Adams EH, Gfroerer JC, Rouse BA, Kazel NJ. Trends in prevalence and consequences of cocaine use. *Adv Alcohol Subst Abuse* 1986; **6**: 49–71.
3. Gawin HF, Ellinwood HE Jr. Cocaine and other stimulants. *N Engl J Med* 1988; **318**: 1173–1181.
4. Mueller PD, Benowitz NL, Olson KR. Cocaine. *Emer Med Clin N Amer* 1990; **8**: 481–493.
5. Riggs D, Weibley RE. Acute toxicity from oral ingestion of crack cocaine: a report of four cases. *Pediatr Emer Care* 1990; **6**: 24–26.
6. Brody SL, Anderson GV, Gutman JB. Pneumomediastinum as a complication of 'crack' smoking. *Am J Emerg Med* 1988; **6**: 241–243.
7. Kussowsky AW, Lyon FA. Cocaine and acute myocardial infarction—a probable connection. *Chest* 1984; **86**: 729–731.
8. Nalbandian H, Sheth N, Dietrich R *et al.* Intestinal ischemia caused by cocaine ingestion: report of two cases. *Surgery* 1985; **96**: 374–376.
9. Murray RJ, Albin RJ, Mergner W *et al.* Diffuse alveolar hemorrhage related to cocaine smoking. *Chest* 1988; **93**: 427–429.
10. Zamora-Quezada JC, Dinerman H, Stadecker MJ *et al.* Muscle and skin infarction after fire basing cocaine (crack). *Ann Intern Med* 1988; **108**: 564–566.
11. Golbe LI, Merkin MD. Cerebral infarction in a user of fire-base cocaine ('crack'). *Neurology* 1986; **36**: 1602–1604.
12. Lee HS, Lamaute HR, Pizzi WF, Picarel SK, Luks FJ. Acute gastroduodenal perforation associated with use of crack. *Ann Surg* 1990; **211**: 15–17.
13. Abramson DL, Kral JG, Gertler JP, Lewis T. Gastropyloric ulcers related to 'crack'. *JAMA* 1989; **262**: 617–618.
14. Abramson DL, Gertler JP, Lewis T, Kral JG. Crack-related perforated gastropyloric ulcer. *J Clin Gastroenterol* 1991; **13**: 17–19.
15. Kram HB, Hardin E, Clark SR, Shoemaker WC. Perforated ulcers related to smoking 'crack' cocaine. *Am Surgeon* 1992; **58**: 293–294.
16. Barefield ES, Xie H. Effect of cocaine on distribution of cardiac output. *Clin Res* 1991; **39**: 866.
17. Stein TA, Keegan LM, Auguste LJ, Bailey B, Wise L. Stress-induced gastric lesions and the synthesis of prostaglandins and leukotrienes. *J Surg Res* 1991; **51**: 368–371.
18. Auguste LJ, Angus LDG, Stein TA, Wise L. Arachidonic acid protection of rat mucosa against stress ulceration. *J Surg Res* 1987; **43**: 103–106.
19. Auguste LJ, Angus LDG, Stein TA, Wise L. Starvation and mucosal prostaglandin-$E_2$ in gastric stress ulcerations. *Critical Care Medicine* 1988; **16**: 610–611.
20. *Guide for the Care and Use of Laboratory Animals*. Institute of Laboratory Animal Resources, National Research Council. Department of Health, Education and Welfare, Publication No. (NIH) 78–23.
21. Brodie DA, Hanson HM. A study of the factors involved in the production of gastric ulcers by the restraint techniques. *Gastroenterology* 1960; **38**: 353.
22. Derlet RW, Albertson TE. Diazepam in the prevention of seizures and death in cocaine-intoxicated rats. *Ann Emer Med* 1989; **18**: 542–546.
23. Stein TA, Angus LDG, Borrero E, Auguste LJ, Wise L. Picogram measurement of prostaglandin $E_2$ synthesis in gastric mucosa by high-performance liquid chromatography. *J Chromatogr* 1987; **385**: 377–382.
24. Stein TA, Bailey B, Auguste LJ, Wise L. Measurement of prostaglandin G/H synthetase and lipoxygenase activity in the stomach wall by HPLC. *Biotechniques* 1991; **10**: 222–225.
25. Jatlow P, Nadim H. Determination of cocaine concentrations in plasma by high-performance liquid chromatography. *Clin Chem* 1990; **36**: 1436–1439.
26. Auguste LJ, Shamash F, Stein TA, Wise L. The effect of sucralfate on the gastric duodenal level of prostaglandin $E_2$. *Current Surg* 1987; **44**: 127–132.
27. Tachi Y, Yang H. Brain regulation of gastric acid secretion by peptides. *Ann NY Acad Sci* 1990; **597**: 128–145.
28. Hernandez DE, Jennes C, Eneerick SG. Brain vasoactive peptide: a potent stimulant of gastric acid secretion. *Brain Res* 1987; **420**: 129–134.
29. Hernandez DE. The role of brain peptides in the pathogenesis of experimental stress gastric ulcer. *Ann NY Acad Sci* 1990; **597**: 28–35.
30. Lauritsen K, Laursen LS, Buklane K, Rask-Madsen J. Effects of topical 5-aminosalicylic acid and prednisolone on prostaglandin $E_2$ and leukotriene $B_4$ levels determined by equilibrium *in vivo* dialysis of rectum in relapsing ulcerative colitis. *Gastroenterology* 1986; **91**: 837–844.
31. Sandor V, Cuparencu B. Analysis of the mechanism of the protective activity of some sympathomimetic amines in experimental ulcers. *Pharmacology* 1977; **15**: 208–217.

**Received 1 June 1993; accepted in revised form 21 July 1993**
